# Associations between childhood behavior and current cognitive and emotional function in adults with irritable bowel syndrome—a cross sectional retrospective study

**DOI:** 10.1186/s12876-026-05067-y

**Published:** 2026-07-02

**Authors:** Julie Billing, Daniela M. Pfabigan, Elisabeth K. Steinsvik, Gülen A. Lied, Birgitte Berentsen, Astri J. Lundervold

**Affiliations:** 1 Department of Clinical and Biological Psychology, University of Bergen, Bergen, Norway; 2 Department of Clinical Medicine, University of Bergen, Bergen, Norway; 3 National Center for Functional Gastrointestinal Disorders, Department of Clinical Medicine, University of Bergen, Bergen, Norway

**Keywords:** Irritable bowel syndrome, Anxiety, Depression, Cognition, Childhood behavior

## Abstract

**Background:**

Childhood behavior may influence emotional regulation and cognitive processing capacities in adulthood. Associations between childhood behavior and present emotional and cognitive functions in adults with Irritable Bowel Syndrome (IBS) are not yet established.

**Objective:**

To explore relationships between retrospectively reported childhood behavior and current emotional and cognitive function in adults with IBS vs healthy controls (HCs).

**Methods:**

Adults with IBS (*n* = 57) and HCs (*n* = 38) completed the Wender Utah Rating Scale (WURS) to assess childhood behavior. Emotional functioning was assessed with the Hospital Anxiety and Depression Scale (HADS), and cognitive functioning with the Repeatable Battery for the Assessment of Neuropsychological Status (RBANS). WURS responses were subjected to principal component analysis (PCA) to identify underlying factors, which were then examined for associations with current emotional and cognitive functioning.

**Results:**

The WURS PCA yielded four factors: (1) Behavioral Self-Regulation and Social Functioning (2) Emotional Reactivity and Temperament, (3) Psychological Vulnerability and Distress, and (4) Academic/Cognitive Challenges. IBS patients scored significantly higher than healthy controls on Factors 3 and 4. Significant correlations were found between Factor 3 and HADS in the HC group, and between Factor 4 and RBANS in the IBS group.

**Conclusions:**

This study demonstrates that IBS patients report more childhood psychological vulnerability and academic/cognitive challenges than controls, and that these retrospectively reported behaviors show distinct associations with adult cognitive functioning. The findings suggest that developmental trajectories, such as neurocognitive traits and early stress-related mechanisms, contribute to the heterogeneity of IBS.

**Trial registration:**

The project is registered at www.clinicaltrials.gov (#NCT04296552), first submitted 2020.03.04.

**Graphical Abstract:**

This study investigates associations between childhood behavior and emotional and cognitive function in adults with IBS. Childhood psychological vulnerability (WURS Factor 3) and academic/cognitive challenges (WURS Factor 4) were notably higher in IBS patients than in healthy controls and demonstrated distinct associations with adult cognitive function (Fullscale RBANS score). *Abbreviations: *IBS: Irritable Bowel Syndrome, WURS: Wender Utah Rating Scale, RBANS: Repeatable Battery for the Assessment of Neuropsychological Function.

**Figure:**
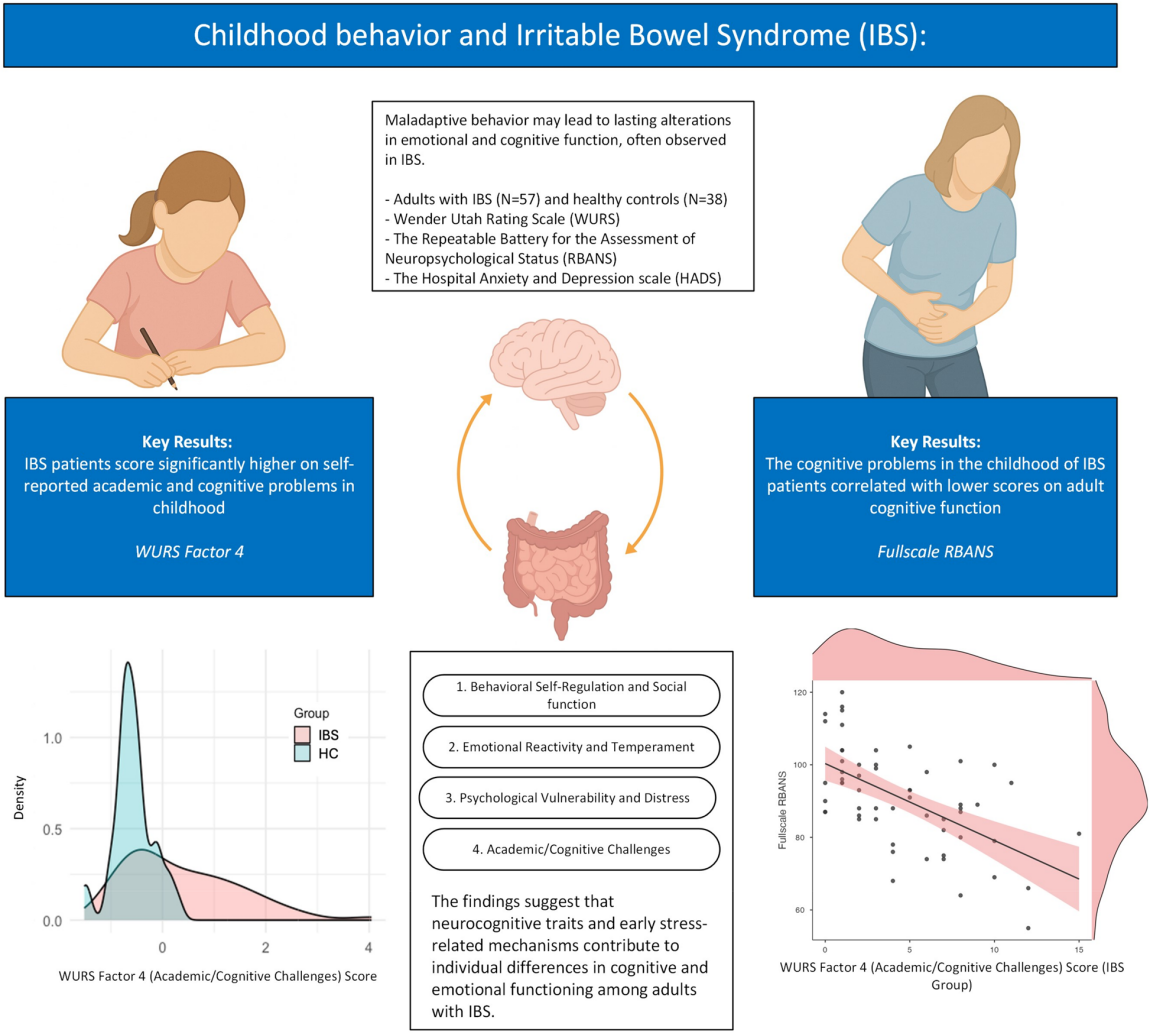

## Background

Irritable bowel syndrome (IBS), one of the most prevalent disorders of gut-brain interaction (DGBI), is increasingly understood as a condition shaped by complex interactions between biological vulnerabilities and psychosocial factors. Some of these factors may have roots in early development. Early life stressors can lead to long-lasting changes in stress-response systems [1–3], and are suggested to affect core features of IBS through a negative impact on the brain-gut axis communication system [4, 5]. Research on early life stress in IBS has predominantly focused on adverse childhood events (ACEs), such as trauma, neglect, and abuse, which are often reported retrospectively by patients in adulthood [6–9] and consistent evidence from both clinical and animal studies indicates that ACEs are linked to increased risk for IBS later in life [10–12]. For example, Bradford et al. [10] used the Early Trauma Inventory Self Report-Form in a large case–control sample (294 IBS patients and 435 controls) and found that IBS patients reported significantly higher rates of general trauma, physical punishment, emotional abuse, and sexual abuse. These results highlight that both ACEs and other non-gastrointestinal symptoms should be systematically assessed in IBS populations.

However, the relationship between ACEs and the development of IBS remains incompletely understood [13]. Not all IBS patients report ACEs, and conversely, not all individuals exposed to ACEs develop IBS. This discrepancy suggests that additional factors, such as individual differences in vulnerability or resilience, may be involved [14]. In this regard, Smith and Pollak [15] highlighted the importance of the child’s characteristics and the child’s perception of stressful events in shaping later outcomes. Supporting this, Talley et al. [16] found that when controlling for neuroticism and psychological comorbidity, the associations between childhood or adult abuse and IBS were no longer significant.

Based on path analysis, they proposed that abuse may induce neuroticism, which in turn predisposes individuals to IBS, rather than abuse acting as a direct causal factor. More broadly, a systematic review of 348 studies identified psychological disorders, stress, and somatic symptoms as consistent risk factors for abdominal pain-related DGBI across children and adults [17]. Notably, child-level behavioral and temperamental characteristics, such as poorer emotional regulation skills, lower self-worth, early hyperactivity, and conduct problems, were also found to influence the persistence of abdominal pain-related DGBI. ACE questionnaires capture exposure to environmental stressors, but do not assess the broader range of childhood behavior that may shape long-term vulnerability. A more comprehensive approach would be to capture the child’s behavioral function, that is, how the child functioned and acted in their early environment, rather than just what happened to them.

This broader behavioral perspective forms the foundation of our current investigation. In one of our recent studies, we have shown that different aspects of emotional and cognitive function are affected in a subgroup of adult patients with IBS [18]. In the present study, we investigated whether these functional aspects of IBS can be traced back to childhood. Because prospective developmental data were not available, we asked participants to retrospectively identify their childhood behavior on the Wender Utah Rating Scale (WURS) [19]. The WURS inquires about symptoms such as behavior problems in school, impulsivity, temper outbursts, emotional lability, and problems with attention. Previous psychometric work has demonstrated that these items can be meaningfully grouped into underlying dimensions. For example, Brevik et al. [20] conducted a factor analysis on the WURS items in a mixed sample including adults with Attention Deficit Hyperactivity Disorder (ADHD) and a control group, and three factors were identified. These factors were named Aggressiveness and Social Problems, Learning and Attention Problems, and Dysthymia, and capture broader behavioral tendencies that may be relevant for understanding developmental pathways in IBS. Aggressive behavior has been linked to gut microbiota composition in several animal studies [21–23], psychological comorbidities such as anxiety and depression are well-documented [24–26], and cognitive differences have been increasingly recognized in adults with IBS [18, 27, 28]. Still, as far as we know, no studies to date have examined potential relationships between childhood behavior, adult IBS, and emotional and cognitive function within an integrated framework. Such an approach could provide insights into the various factors associated with IBS and its comorbidities.

The objectives of this study are to characterize childhood behavior and explore associations between these characteristics and current emotional and cognitive function. Our analytic approach includes a Principal Component Analysis (PCA) performed on the WURS items. The factors generated from the PCA are used to analyze the differences between patients with IBS and healthy controls (HCs), and to examine associations between childhood behavior and current measures of emotional and cognitive function.

## Methods

This study was designed and reported in accordance with the STROBE (Strengthening the Reporting of Observational Studies in Epidemiology) guidelines to ensure transparency and completeness in reporting observational research [29].

### Irritable Bowel Syndrome Symptom Severity Scale

The Irritable Bowel Syndrome Symptom Severity Scale (IBS-SSS) is a self-report questionnaire that was included to characterize the severity level of IBS of the included participants. The scale includes questions about abdominal pain severity, abdominal pain frequency, abdominal bloating, satisfaction with bowel habits, and interference with daily life [30]. Each individual item is scored from 0 (no symptoms/no interference) to 100 (worst imaginable symptoms/complete interference), with patients marking their response on a 100 mm visual analog scale that gets converted to the 0–100 point score. The total score ranges from 0 to 500, where < 75 is defined as normal/no IBS, 75–175 is defined as mild IBS, 175–300 is defined as moderate IBS, and a total score of ≥ 300 is defined as severe IBS [30]. For this study, inclusion in the IBS group required an IBS-SSS score of > 175. Scores in the control group predominantly fell within the normal range (IBS-SSS score < 75). Three HCs with scores slightly above this threshold were nonetheless retained in the analyses, as their scores on the primary study measures were close to the median of the total HC group.

### The Wender Utah Rating Scale

The Wender Utah Rating Scale (WURS) is a self-report questionnaire in which the participants are asked to rate a set of 25 items describing different aspects of childhood behavior and emotions [19]. The questionnaire is introduced by: “As a child I was (or had):” to ensure retrospective assessment of childhood behaviors. Then participants are asked to rate each item on a five-point Likert scale: 0 = “not at all or very slightly”, 1 = “mildly”, 2 = “moderately”, 3 = “quite a bit” or 4 = “very much”. The scale was originally designed to assess behavior typical of children with ADHD, but research shows that its utility extends beyond ADHD (see e.g., [31]). The total WURS score is the sum of responses across the 25 items, with higher scores reflecting more severe problems.

### The Hospital Anxiety and Depression Scale

The Hospital Anxiety and Depression Scale (HADS) is a self-report questionnaire that was originally developed and validated for use in non-psychiatric hospital settings to screen for anxiety and depression [32]. In that anxiety and depression are recognized as core dimensions of emotional functioning, HADS scores are used in the present study as an operationalization of this construct. Participants are asked to evaluate their experiences during the previous week. HADS includes 14 items, 7 for anxiety and 7 for depression, where each item is scored 0–3, with response options varying by question (e.g.,”not at all” to”most of the time” or”definitely” to”not at all”), with a maximum total score of 42 (21 on each subscale). The participants can be categorized as either a non-case (< 8), a doubtful/borderline case (8–10) or a case (≥ 11) [32]. Participants completed the HADS questionnaire online during the same week they underwent the cognitive assessment.

### The Repeatable Battery for the Assessment of Neuropsychological Status

The Repeatable Battery for the Assessment of Neuropsychological Status (RBANS) [33] is a cognitive test battery with ten subtests that generates the following five index scores: immediate memory, visuospatial abilities, verbal skills, attention, and recall [33]. A total (Fullscale) index score is computed based on performance across all subtests. In the present study, we will report and analyze scores that are standardized according to age-adjusted Scandinavian norms [34], with a mean score of 100, with one standard deviation (SD) equals 15 points. Higher scores indicate better cognitive functioning. All participants completed the A-version of the Norwegian version of the RBANS according to standardized procedures outlined in the test manual [34]. The assessment typically requires approximately 20–30 min to complete.

### Self-reported GI symptoms in childhood

A subsample of the participants was asked a single yes/no question concerning childhood gastrointestinal (GI) problems (*n* = 88): “Did you experience gastrointestinal problems in childhood?” Although not as comprehensive as validated measures of ACE, it provides a direct retrospective marker of whether participants recall recurring GI problems during development. Responses were coded as binary (yes/no) and analyzed using chi-square tests and odds ratios to compare the prevalence of reported childhood GI problems between the IBS and HC groups.

### Participants

The participants were part of the multidisciplinary Bergen Brain-Gut Microbiota (B-BGM) project, conducted at Haukeland University Hospital in Bergen, Norway, between 2019 and 2021. In total, 128 participants were included in the original study. Approximately half of the IBS patients were recruited via the hospital’s outpatient clinic, while the remaining patients and all HCs were recruited through media advertisements and flyers. Participants were directed to review the detailed study information that was available on the project’s website, followed by a telephone interview with a study nurse. IBS patients were screened using predefined inclusion and exclusion criteria (see Table 1). They were also offered optional clinical evaluations, including ultrasonography, sigmoidoscopy, upper endoscopy, and blood tests. HCs were required to be over 18 years of age and were screened using the same inclusion and exclusion criteria as the IBS group. For a more detailed description of the study protocol and methodology, see Berentsen et al. [35].

**Table 1:** Exclusion and inclusion criteria for patients with IBS

Inclusion	Meet Rome-IV criteria: reporting recurrent abdominal pain on average at least 1 day per week during the last 3 months, and associated alteration in bowel habits with duration for at least 6 months prior to diagnosis IBS-SSS score above 175 Normal (Norwegian) diet for the last 3 weeks before inclusion
Exclusion	No pharmacological treatment affecting the GI tract, including medication for anxiety and depression No presence of other organic diseases such as diabetes, coeliac disease, IBD, Polycystic ovary syndrome, active Helicobacter pylori infection, Parkinson’s disease, amyotrophic lateral sclerosis, or a psychiatric disorder dependent on pharmacological treatment with known gastrointestinal side-effects Treated with antibiotics for the last 3 months Diets such as vegetarian or vegan Usage of probiotics or low-FODMAP diet within the last 3 weeks Pregnancy Previous intestinal surgery, except appendectomy Metallic implants not compatible with MRI, or claustrophobia Travel outside Europe for the last 3 weeks or plan to travel in the near future

Source: Retrieved from and pre-specified in the Bergen Brain-Gut-Microbiota (B-BGM) study protocol [35]

*Abbreviations: IBS* Irritable Bowel Syndrome, *IBS-SSS* IBS Symptom Severity Score, *IBD* Inflammatory Bowel Disease, *MRI* Magnetic Resonance Imaging, *GI* Gastrointestinal, *low-FODMAP* low Fermentable Oligosaccharides Disaccharides Monosaccharides and Polyols diet

The target sample size for the overall B-BGM study was pre-specified in the study protocol as 100 IBS patients and 40 healthy controls, based on an anticipated medium effect size (f^2^ = 0.20), a desired statistical power of 80%, and a type I error rate of 5%, yielding a minimum required sample size of 59 per group for multiple regression analyses [35]. The sample size was further increased to 100 IBS patients to allow for diagnostic subclassification and to account for anticipated missing data. However, recruitment was substantially affected by the COVID-19 pandemic, which disrupted data collection between 2019 and 2021, resulting in a final sample of 57 IBS patients and 38 healthy controls for the primary WURS analyses. 

**Fig. 1:**
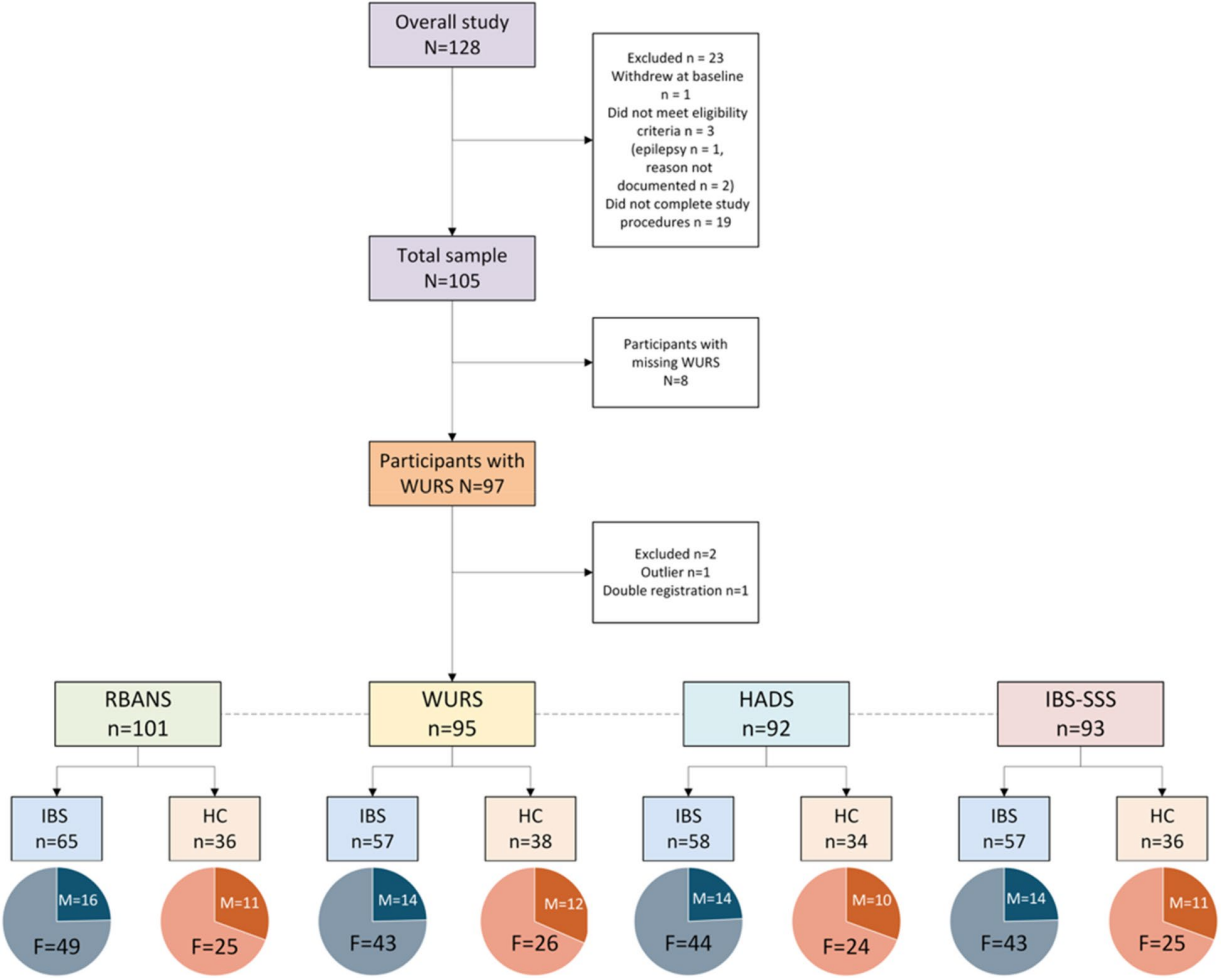
STROBE participant flow chart of the number of participants included in this study. *Abbreviations:* IBS: Irritable Bowel Syndrome, HC: Healthy Controls, WURS: Wender Utah Rating Scale, HADS: Hospital Anxiety and Depression Scale, RBANS: Repeatable Battery for the Assessment of Neuropsychological Status, IBS-SSS: IBS Severity Scoring System

Of the 128 participants included in the B-BGM study, the numbers who completed the different questionnaires and tests were different. The numbers included in the analyses will therefore differ. Figure 1 shows the numbers and sex distributions for each of the measures in the IBS and HC groups. Of the 97 participants who filled out the WURS questionnaire, one participant had three missing responses. To account for these missing values, we imputed them using the mean of the participant’s non-missing scores. In the same sample, we found one participant with an extremely high total WURS score (T = 82). This participant was excluded from further analysis. During data cleaning, we identified one participant who had been entered twice, with discrepant WURS scores. We cross-checked the original files and removed the incorrect duplicate. Thus, the WURS sample included *N* = 95 participants.

### Data analysis

To reduce dimensionality and uncover latent behavioral constructs, a PCA with varimax rotation was conducted on the WURS items. This data-driven approach enabled the identification of coherent behavioral domains relevant to our sample of IBS patients and healthy controls. PCA was conducted on the full sample (*N* = 95, IBS *n* = 57 and HCs *n* = 38) with WURS scores to maximize variance and identify generalizable behavioral constructs. This approach ensured comparability of factor scores across groups and provided stable components for subsequent group comparisons and correlation analyses. To evaluate the quality of the current PCA, we conducted the Kaiser–Meyer–Olkin (KMO) test and Bartlett’s test of sphericity. The overall KMO measure of sampling adequacy was 0*.*796, exceeding the recommended threshold of 0*.*70 [36], with all individual items showing values > 0*.*62. Bartlett’s test of sphericity was significant, *χ*^2^(300) = 1383, *p* < *0.0*01, indicating that the correlation matrix was appropriate for principal component analysis [37].

The R package psych was used in descriptive analyses of the clinical characteristics of the total sample and within the two groups (IBS and HC) [38]. The PCA was conducted in *jamovi* (Version 2.6; The jamovi project, 2024) [39], which implements the principal function from the psych package [38]. The two groups were compared with Welch’s t-tests to account for unequal variances and the Mann–Whitney U test for non-normally distributed data. Effect sizes were interpreted using Cohen’s *d*, where values around 0.2 are considered small, 0.5 medium, and 0.8 large. Spearman’s rho correlation coefficients were calculated to examine associations between the four WURS factors and continuous outcome variables, including HADS total, anxiety, and depression subscores, RBANS Fullscale and the RBANS indices. Correlations were computed using the cor.test function in R [40], with pairwise deletion for missing data. Heatmaps were created using ggplot2 [41]. Scatterplots were generated in *jamovi* (Version 2.6; The jamovi project, 2024) [39]. When appropriate, group-specific correlations (IBS and HC separately) were also explored. Given that the four *WURS* factors represent conceptually distinct predictors derived from the same questionnaire, Bonferroni correction was applied across these four factors (*α*_adj_ = *0.0*5*/*4 = *0.0*125). This approach controls for family-wise error across the predictors rather than across all individual correlations (*n* = 40), reflecting our theoretical focus on the *WURS* dimensions. Accordingly, in the Results section, we report only correlations that remained significant after applying this corrected threshold. For correlations that were significant in only one group, we compared their magnitudes using Fisher’s *r*-to-*z* tests (two-tailed).

## Results

### Characteristics of the participants

In the WURS sample (*N* = 95), the mean age was 37.2 (SD = 11.6) years and the mean education was 16 (SD = 1.84) years, with non-significant differences between the IBS and the HC groups (*p* = 0.510 and *p* = 0.303, respectively).

Table 2 shows that the IBS group is significantly more impaired on the IBS-SSS, HADS, the Immediate Memory index, the Recall index, the Fullscale RBANS score and the total WURS score. The largest effect size was observed for IBS symptom severity (IBS-SSS) (*d* = 3.90). This large group difference was expected, as inclusion criteria required IBS patients to have an IBS-SSS score > 175, whereas healthy controls did not report clinically significant symptoms. Emotional symptoms (HADS) also showed significant group differences, with depression and anxiety with a medium effect size, and HADS total score with a large effect size. Group differences in cognitive performance were overall smaller (negligible to medium), with the largest effect for the RBANS Fullscale. A medium to large effect was observed on the total WURS score.

**Table 2:** Results of the IBS and HC group

Measure	IBS Mean (SD)	HC Mean (SD)	*t* / *U*	*p*	*d* (95% CI)
**Emotion (HADS)**	(*n* = 58)	(*n* = 34)			
Depression^1^	4.62 (3.1)	2.12 (2.4)	494	<.001***	0.88 [0.43, 1.31]
Anxiety^1^	8.03 (4.2)	4.18 (3.4)	470	<.001***	1.02 [0.54, 1.43]
Total^1^	12.7 (6.3)	6.29 (5.4)	414	<.001***	1.08 [0.61, 1.51]
**Cognition (RBANS)**	(*n* = 65)	(*n* = 36)			
Immediate Memory	89.8 (16.1)	98.6 (15.8)	2.68	.009**	–0.55 [–0.97, –0.14]
Visuospatial^1^	94.1 (11.7)	95.1 (12.3)	1104	.639	–0.07 [–0.48, 0.33]
Verbal Skills	97.6 (14.4)	101.4 (13.8)	1.28	.203	–0.26 [–0.67, 0.15]
Attention^1^	93.0 (12.2)	99.8 (18.5)	994	.213	–0.46 [–0.87, –0.05]
Recall^1^	93.6 (15.1)	102.4 (19.8)	741	.002**	–0.52 [–0.93, –0.11]
Fullscale	90.7 (13.1)	99.7 (12.7)	3.36	.001**	–0.69 [–1.11, –0.27]
**IBS Severity**	(*n* = 57)	(*n* = 36)			
IBS-SSS^1^	271 (73.8)	34.4 (29.5)	0	<.001***	3.90 [3.19, 4.59]
**Childhood Behavior**	(*n* = 57)	(*n* = 38)			
Total WURS^1^	23.2 (12.6)	15.2 (11.2)	623	<.001***	0.66 [0.24, 1.08]

Between-group differences in emotional functioning, cognitive performance, IBS severity, and retrospectively assessed childhood behavior

Group comparisons were conducted using Welch’s independent samples t-test, except where indicated

*Abbreviations: WURS* Wender Utah Rating Scale, *HADS* Hospital Anxiety and Depression Scale, *RBANS* Repeatable Battery for the Assessment of Neuropsychological Status, *IBS-SSS* IBS Severity Scoring System, *IBS* Irritable Bowel Syndrome, *HC* Healthy Controls, *SD* Standard deviation

^1^Mann–Whitney U test used due to non-normality (Shapiro–Wilk). Cohen’s *d* is reported as the effect size for all comparisons

### Principal component analysis (PCA)

A PCA with varimax rotation with an eigenvalue > 1 initially generated a six-factor solution, explaining 70.3% of the variance. With factor loadings greater than 0.5, the fifth and sixth factors only contained three and one item(s), respectively. With a fixed number of three components and factor loadings greater than 0.4, three factors explained 53,1% of the variance. With a fixed number of four components and factor loadings greater than 0.4, four factors explained 60.3% of the variance. The four-factor model was selected because it demonstrated acceptable explained variance (60.3%) and generated factors that were clinically meaningful and consistent with theoretical expectations (Table 3). Based on their content, the factors were labeled as follows: Factor 1, *Behavioral Self-Regulation and Social Functioning*; Factor 2, *Emotional Reactivity and Temperament*; Factor 3, *Psychological Vulnerability and Distress*; and Factor 4, *Academic/Cognitive Challenges*. The items included in each factor are presented in Table 3.

**Table 3:** Factor loadings of childhood behavioral characteristics (WURS items)

As a child I was (or had)	Factor 1	Factor 2	Factor 3	Factor 4
**Behavioral Self-Regulation and Social Functioning**				
Q15: Trouble seeing things from someone else’s point of view	0.775			
Q16: Acting without thinking	0.769			
Q4: Inattentive, daydreaming	0.727			
Q22: Trouble with authorities, trouble with school	0.673			
Q21: Unpopular with other children	0.611			
Q19: Losing control of myself	0.611	0.421		
Q7: Trouble with stick-to-it-iveness	0.587			
Q10: Disobedient with parents, rebellious, sassy	0.575			
**Emotional Reactivity and Temperament**				
Q5: Hot- or short-tempered, low boiling point		0.861		
Q6: Temper outbursts, tantrums		0.803		
Q14: Angry		0.793		
Q12: Irritable		0.749		
Q13: Moody, ups and downs		0.666		
Q8: Stubborn, strong-willed		0.551		
Q18: Guilty feelings, regretful	0.452	0.528		
**Psychological Vulnerability and Distress**				
Q2: Anxious, worrying			0.799	
Q17: Tendency to be immature			0.766	
Q3: Nervous, fidgety			0.713	
Q9: Sad or blue, depressed, unhappy			0.703	
Q11: Low opinion of myself			0.692	
Q20: Tendency to be or act irrational			0.549	
**Academic/Cognitive Challenges**				
Q24: Trouble with mathematics or numbers				0.834
Q23: Overall a poor student, slow learner				0.812
Q25: Not achieving up to potential				0.733
Q1: Concentration problems, easily distracted	0.559			0.586

This table presents the factor loadings on each WURS item from the Principal Component Analysis (PCA). Four distinct factors emerged, named (1) Behavioral Self-regulation and Social Functioning, (2) Emotional Reactivity, (3) Psychological Vulnerability and Distress, and (4) Academic/Cognitive Challenges. Loadings below 0.40 are omitted for clarity. The PCA was conducted using varimax (orthogonal) rotation. A sensitivity analysis using promax (oblique) rotation, which permits correlated factors, yielded identical item-to-factor assignments across all four components (inter-component correlations ranging from 0.09 to 0.39), supporting the robustness of the reported factor structure

*Abbreviations: WURS* Wender Utah Rating Scale

Orthogonal (uncorrelated) components are assumed in the varimax rotation. Inspection of the factor intercorrelations revealed that some WURS factors were moderately correlated, suggesting the independence assumption was not fully met. To assess the robustness of the factor solution, we conducted a sensitivity analysis using promax oblique rotation, which permits correlated factors. Inter-component correlations after promax rotation ranged from −0.09 to 0.39, and the assignment of items to factors 1–4 was identical to that obtained with varimax rotation. This finding is consistent with Costello and Osborne [42], who noted that orthogonal and oblique rotations produce comparable results when factors are only moderately correlated. We therefore retain the varimax solution in the main analyses.

Internal consistency of the WURS items was assessed using Cronbach’s *α* and McDonald’s *ω*. The overall scale demonstrated good reliability (Cronbach’s *α* = 0*.*880, McDonald’s *ω* = 0*.*891), both exceeding the conventional threshold of 0*.*70 [43, 44]. Reliability analyses were also conducted separately for each of the identified factors, which all showed acceptable to good internal consistency (Factor 1: Cronbach’s *α* = 0*.*847, McDonald’s *ω* = 0*.*851, Factor 2: Cronbach’s *α* = 0*.*861, McDonald’s *ω* = 0*.*881, Factor 3: Cronbach’s *α* = 0*.*778, McDonald’s *ω* = 0*.*780, Factor 4: Cronbach’s *α* = 0*.*793, McDonald’s *ω* = 0*.*808). These findings support the adequacy of the data for PCA and the internal reliability of the derived factors.

Regarding the WURS factors, we found that the two groups were not significantly different on the first two factors (*Behavioral Self-Regulation and Social Functioning* and *Emotional Reactivity and Temperament*), while differences with moderate to large effect sizes were observed for Factor 3 (*Psychological Vulnerability and Distress*) and Factor 4 (*Academic/Cognitive Challenges*) (see Table 4).

**Table 4:** Between-group comparisons of childhood behavior (WURS Factor scores)

Childhood behavior factors	**Mean (SD)**		*U*	*p*	*d* (95%CI)
	IBS (*n* = 57)	HC (*n* = 38)			
**WURS Factor 1**					
Behavioral Self-Regulation & Social Functioning	5.67 (5.2)	4.84 (4.9)	961	0.351	0.16 [–0.25, 0.57]
**WURS Factor 2**^1^					
Emotional Reactivity & Temperament	6.67 (5.7)	5.11 (3.9)	947	0.302	0.31 [–0.10, 0.72]
**WURS Factor 3**^1^					
Psychological Vulnerability & Distress	6.37 (4.6)	3.97 (4.3)	690	0.003***	0.54 [0.12, 0.95]
**WURS Factor 4**^1^					
Academic/Cognitive Challenges	4.46 (3.8)	1.32 (1.4)	492	<.001***	1.05 [0.61, 1.48]

Mean differences in WURS factor scores between IBS patients and HCs. Higher scores reflect a higher number of retrospectively reported maladaptive childhood behaviors

*Abbreviations: WURS* Wender Utah Rating Scale

^1^Mann–Whitney U test used due to significant non-normality (Shapiro–Wilk). The *U* column reports the Mann– Whitney U statistic for all factors

A significantly larger proportion of participants in the IBS group (32 of 52; 61.5%) reported having had gastrointestinal (GI) problems in childhood compared to healthy controls (8 of 36; 22.2%), *χ*^2^(1*,N* = 88) = 13*.*3, *p* < 0.001. The odds of reporting childhood GI problems were nearly six times higher in the IBS group (OR = 5.60, 95% CI [2.14, 14.7]) than in the HC group, indicating that patients with IBS were substantially more likely to recall such symptoms from their childhood than HCs. Within the IBS group, however, comparisons between those who reported childhood GI problems (*n* = 32) and those who did not (*n* = 20) revealed no significant differences in any of the outcome measures, including RBANS, HADS, and WURS factors.

### Correlations between cognitive and emotional function in childhood and adulthood

To examine associations between childhood behavior and cognitive and emotional function in adulthood, we conducted correlation analyses using Spearman’s rho. This non-parametric test was chosen due to non-normal distributions of the variables, as indicated by the Shapiro–Wilk tests. Correlations with anxiety/depression (HADS) and cognitive performance scores (RBANS) were calculated separately for each of the WURS factors.

### WURS factors and RBANS

#### Whole sample

In the sample with complete scores on both the RBANS and WURS (*n* = 91), higher scores on *WURS Factor 4 (Academic/Cognitive Challenges)* were associated with poorer overall cognitive performance, as reflected by a statistically significant negative correlation with the RBANS Fullscale (*r*_*s*_ = − 0*.*529, *p* < 0.001). Factor 4 was also significantly correlated with the Immediate Memory Index (*r*_*s*_ = − 0*.*532, *p* < 0.001), the Attention (*r*_*s*_ = − 0*.*344, *p* < 0.001), and Recall (*r*_*s*_ = − 0*.*362, *p* < 0.001) indices. A less strong, but still statistically significant, correlation was found between *WURS Factor 3 (Psychological Vulnerability and Distress)* and the RBANS Fullscale (*r*_*s*_ = − 0*.*262, *p* = 0.012).

#### Within the IBS and the HC groups

**Fig. 2:**
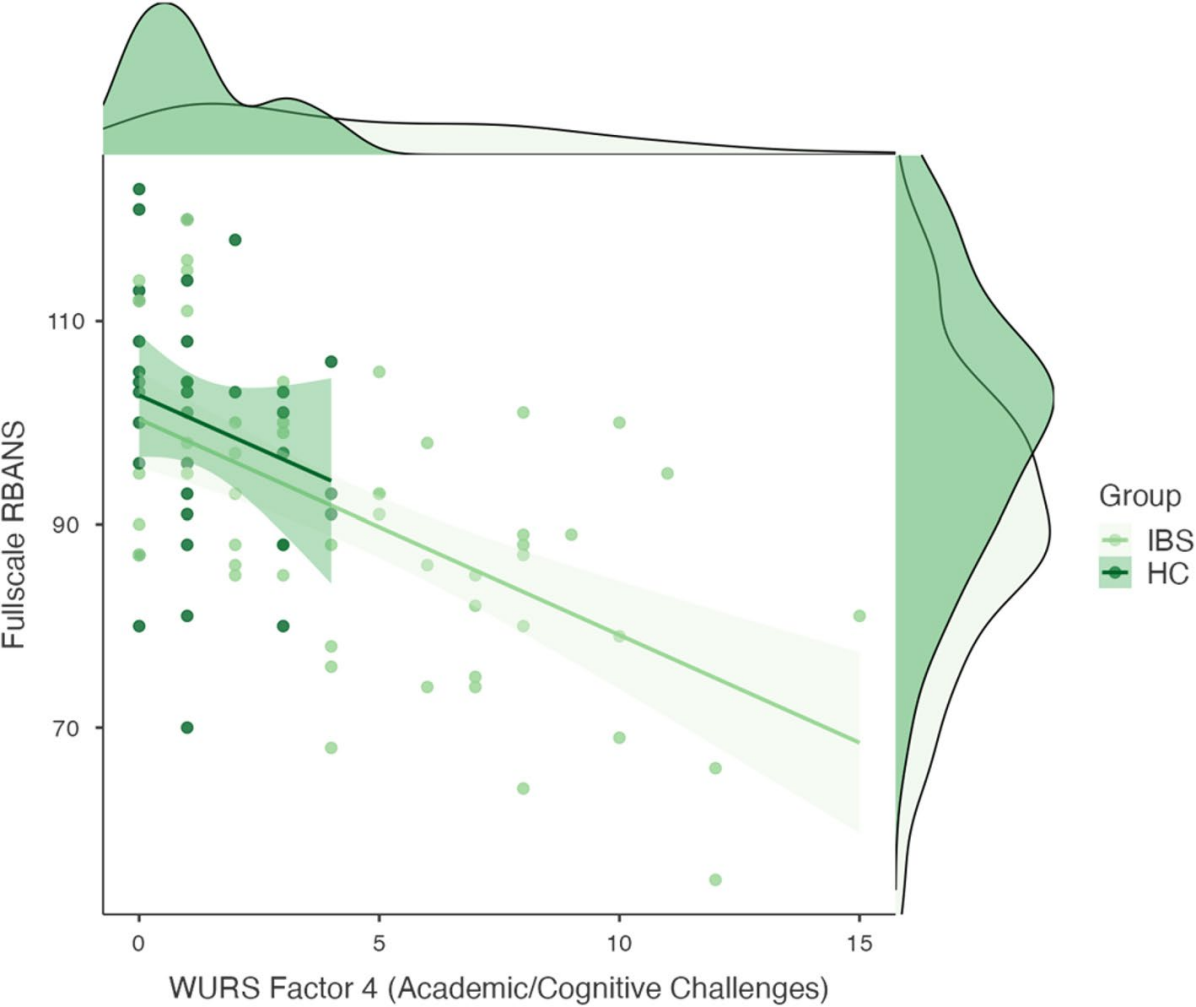
Scatterplot showing the association between RBANS Fullscale score and WURS Factor 4 (Academic/Cognitive Challenges) for the IBS and HC groups. *Abbreviations:* IBS: Irritable Bowel Syndrome; HC: healthy controls; WURS: Wender Utah Rating Scale; RBANS: Repeatable Battery for the Assessment of Neuropsychological Status

**Fig. 3:**
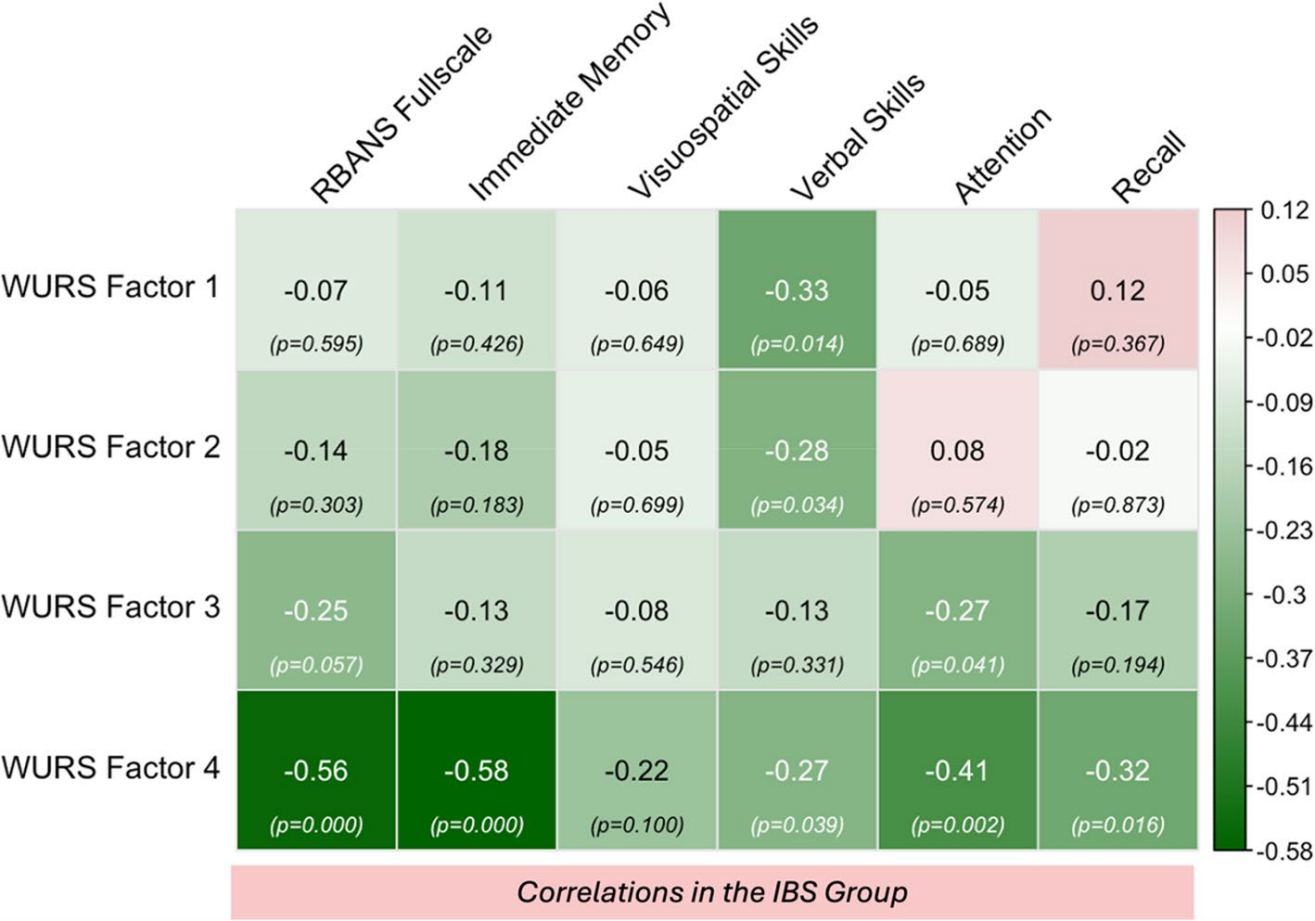
Correlation matrix showing the correlation coefficients (r_s_) and corresponding *p*-values for associations between WURS factors and RBANS domains in the IBS group. *Abbreviations:* WURS: Wender Utah Rating Scale; RBANS: Repeatable Battery for the Assessment of Neuropsychological Status

**Fig. 4:**
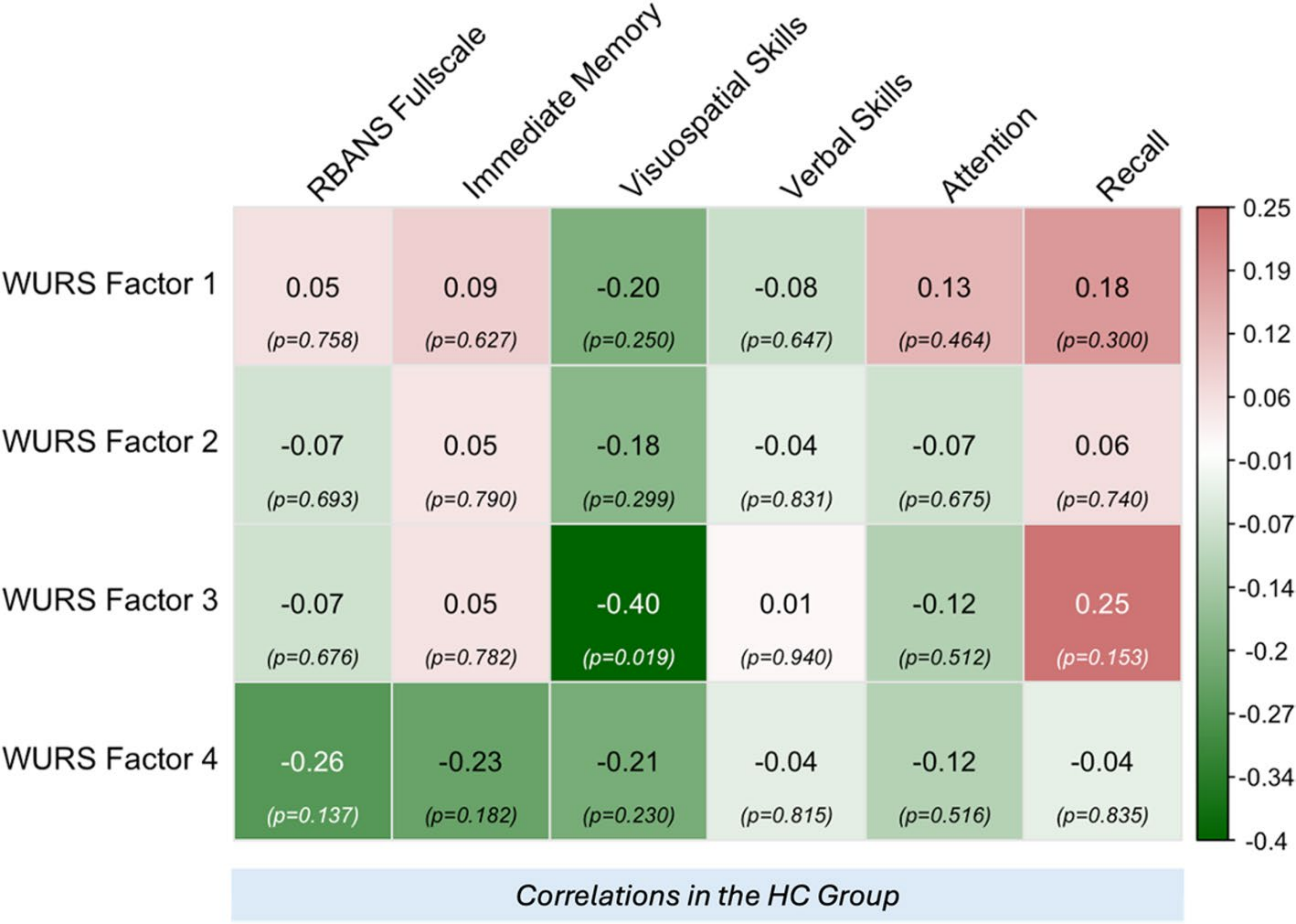
Correlation matrix showing the correlation coefficients (*r*_*s*_) and corresponding *p*-values for associations between WURS factors and RBANS domains in the healthy control group. *Abbreviations:* WURS: Wender Utah Rating Scale; RBANS: Repeatable Battery for the Assessment of Neuropsychological Status

Several significant correlations were observed between childhood difficulties and adult cognitive performance in the IBS group (*n* = 57). The *WURS Factor 4 (Academic/Cognitive Challenges)* was significantly correlated with the RBANS Fullscale (*r*_*s*_ = − 0*.*556, *p* < 0.001) (see Fig. 2), the Immediate Memory (*r*_*s*_ = − 0*.*575, *p* < 0.001) and Attention (*r*_*s*_ = − 0*.*411, *p* = 0.002) indices (see Fig. 3). None of the correlations were statistically significant after Bonferroni correction in the HC group (see Fig. 4).

### WURS factors and HADS

#### Whole sample

A medium-sized positive association was found between *WURS Factor 3 (Psychological Vulnerability and Distress)* and the HADS total score (*r*_*s*_ = *0.5*22, *p* < 0.001) in the sample with scores on both the HADS and WURS (*n* = 86). The correlation was at a similar level both for depressive symptoms (*r*_*s*_ = *0.4*54, *p* < 0.001) and anxiety (*r*_*s*_ = *0.4*68, *p* < 0.001). *WURS Factor 4 (Academic/Cognitive Challenges)* was significantly correlated with the depression (*r*_*s*_ = *0.2*84, *p* = 0.008) score and the total HADS score (*r*_*s*_ = *0.3*05, *p* = 0.004), though the strengths of the associations were somewhat weaker.

#### Within the IBS and the HC groups

**Fig. 5:**
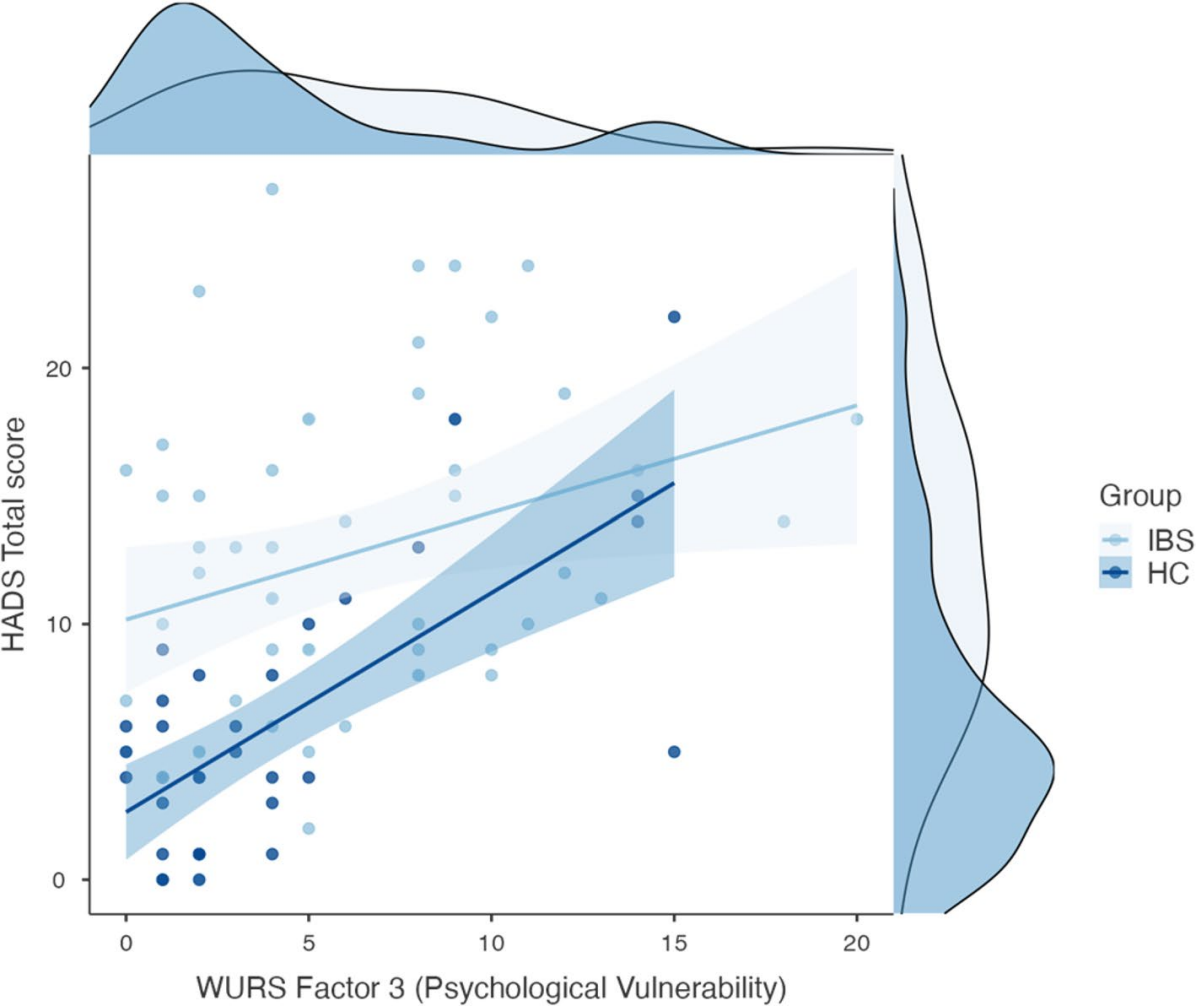
Scatterplot showing the association between HADS Total score and WURS Factor 3 (Psychological Vulnerability and Distress) in the IBS and HC groups. *Abbreviations:* IBS: Irritable Bowel Syndrome; HC: healthy controls; WURS: Wender Utah Rating Scale; HADS: Hospital Anxiety and Depression Scale; IBS-SSS: IBS Severity Scoring System

**Fig. 6:**
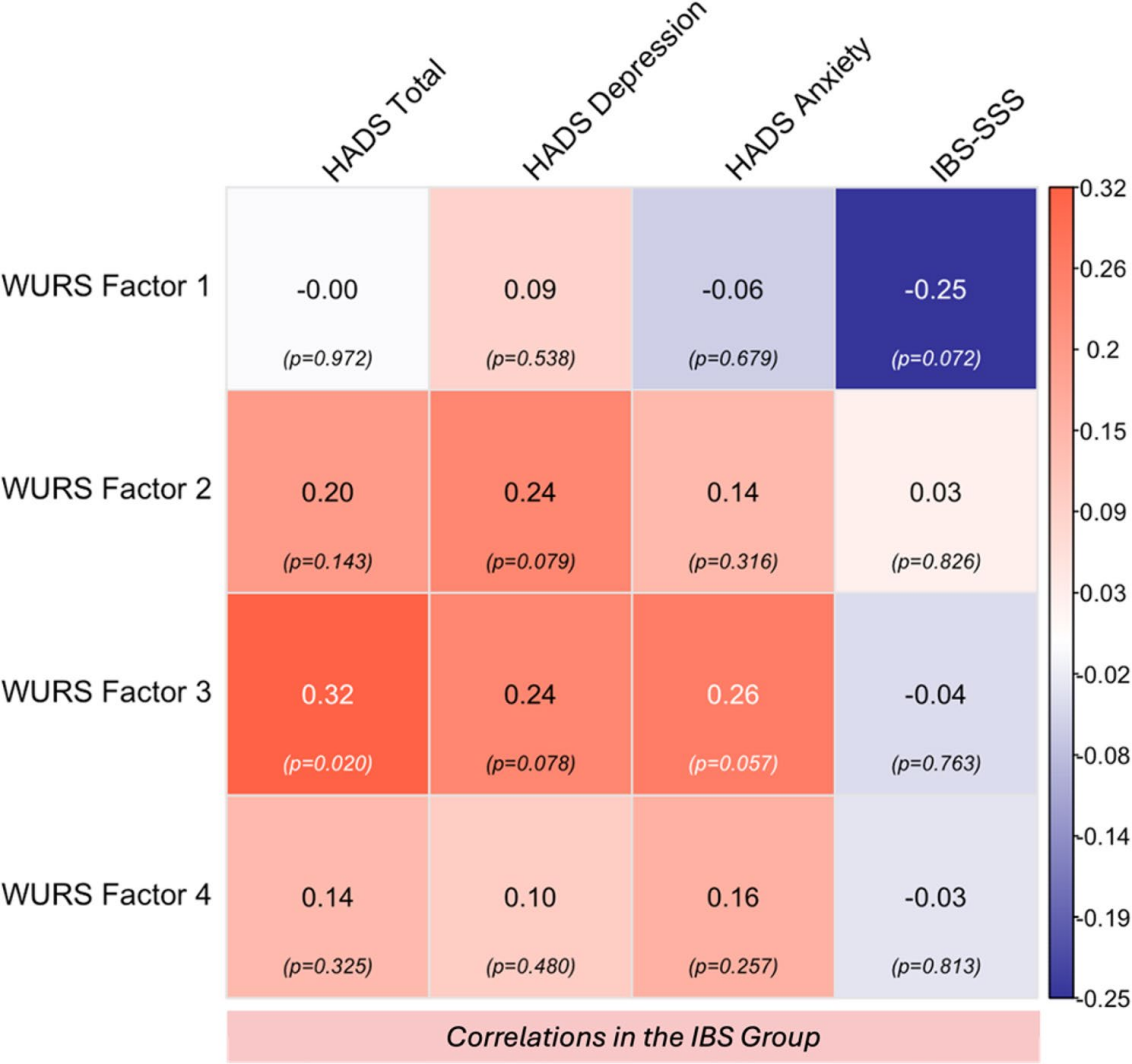
Correlation matrix showing correlation coefficients (r_s_) and corresponding p-values for associations between WURS factors, HADS, and IBS–SSS in the IBS group. Abbreviations: WURS: Wender Utah Rating Scale; HADS: Hospital Anxiety and Depression Scale; IBS-SSS: IBS Severity Scoring System

**Fig. 7:**
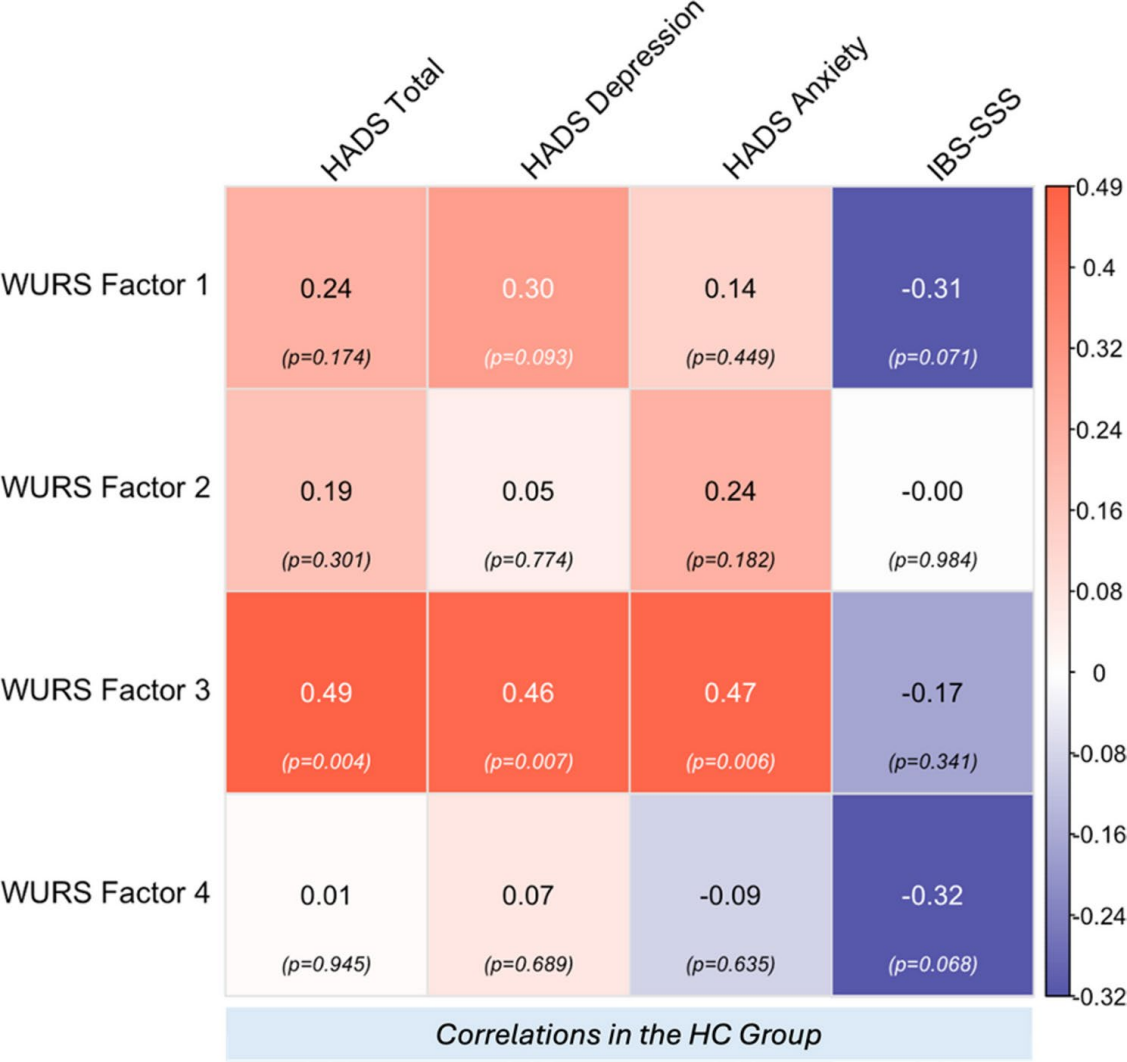
Correlation matrix showing correlation coefficients (r_s_) and corresponding p-values for associations between WURS factors, HADS, and IBS–SSS in the healthy control group. Abbreviations: WURS: Wender Utah Rating Scale; HADS: Hospital Anxiety and Depression Scale; IBS-SSS: IBS Severity Scoring System.

In the HC group (*n* = 33), the correlations between *WURS Factor 3 (Psychological Vulnerability and Distress)* and the HADS scores were rather strong: higher childhood psychological vulnerability and distress was associated with more severe depressive symptoms (*r*_*s*_ = *0.4*58, *p* = 0.007) and higher anxiety (*r*_*s*_ = *0.4*69, *p* = 0.006) and Total HADS score (*r*_*s*_ = *0.4*85, *p* = 0.004) (see Fig. 5). No other WURS factor correlated significantly with HADS scores in the HC group (see Fig. 6). In the IBS group (*n* = 53), the correlations were more modest, and none survived Bonferroni correction (see Fig. 7).

#### Between-group comparisons of correlations

Correlations that were significant in only one of the groups were compared according to the Fisher’s *r*-to-*z* test (two-tailed). The *WURS Factor 4*–RBANS Fullscale correlation was stronger in magnitude in the IBS (*r*_*s*_ = − *0.5*56, *n* = 57) than the HC group (*r*_*s*_ = − *0.2*60, *n* = 34), although this difference did not reach statistical significance (*z* = − 1*.*63, *p* = 0.103). Overall, several associations were consistently observed in the expected direction across groups, with associations generally stronger in the IBS group. While formal comparisons did not yield significant differences, this convergent pattern suggests that childhood cognitive difficulties may be more strongly associated with adult cognitive performance in IBS patients than in healthy controls.

#### Retrospective sensitivity analysis

As this study was not able to recruit the planned number of participants due to the COVID-19 pandemic, a retrospective sensitivity analysis was conducted to evaluate the statistical power of the reported analyses. For the between-group comparisons (*t-*tests), the sample comprised 57 IBS patients and 38 healthy controls (HCs). With this sample size, an effect size of *d* > 0*.*60 can be detected with statistical power larger than 80%, indicating adequate power to detect medium-to-large effects. Thus, comparisons involving WURS Factor 4 were well-powered, whereas those for Factor 3 were slightly underpowered.

For the correlation analyses, the smallest total sample (*N* = 86) provided sufficient power to detect medium-sized correlations (*r* = 0*.*30) at 0.80 power. When analyzed by group, the IBS subsample (*N* = 53) retained sufficient power to detect correlations *r* ≥ 0*.*37, whereas the smaller HC group (*N* = 33) had sufficient power to detect correlations *r* ≥ 0*.*45. These estimates suggest that the study was sufficiently powered to detect medium-to-large-sized associations, while smaller effects could not be reliably described.

## Discussion

In this study, we explored childhood behavioral characteristics of patients with IBS and a group of HCs, as well as associations between these characteristics and cognitive and emotional functioning in adulthood. Our results indicate distinct patterns of association between retrospectively reported childhood behavior and current psychological functioning in IBS patients versus HCs. While HCs demonstrated typical developmental continuity, where childhood psychological distress correlated with adult emotional problems, IBS patients showed a pattern where childhood academic difficulties, more specifically, were linked to adult cognitive impairment. These seemingly divergent patterns suggest that gastrointestinal symptoms or chronic pain in IBS may alter psychological development, highlighting the need to consider childhood behavioral factors when understanding cognitive comorbidities in this population.

### Strengths and limitations

To our knowledge, this is the first investigation to examine a broad range of childhood behavioral characteristics in patients with IBS and relate them to adult cognitive and emotional functioning. By using PCA-derived WURS factors, the study moves beyond traditional group comparisons and highlights specific retrospectively reported childhood patterns that may contribute to the psychological variability observed within the IBS population, consistent with results from prior studies showing clinically heterogeneous subgroups [45]. Another strength is the inclusion of multiple validated outcome measures (RBANS and HADS), which allowed us to explore both cognitive and emotional domains in relation to retrospective childhood reports.

Several limitations should be considered when interpreting these findings. First, the cross-sectional and retrospective design precludes causal inference, and developmental conclusions should be drawn with caution. Second, the sample size was modest, particularly in the HC group (*n* = 38), resulting in low statistical power for detecting small effects.

The inclusion of both groups in a joint PCA carries the advantage of capturing the full range of symptom variation, but may obscure group-specific factor structures. Future studies with larger samples should consider running separate PCAs per group.

Regarding selection bias, participants were recruited from a tertiary outpatient clinic and may not represent the broader IBS population. Sex and age were not formally included as covariates in the correlation analyses, and residual confounding cannot be excluded; studies with larger and more sex-balanced samples should examine whether associations differ by sex. Retrospective self-report is subject to memory bias, though impaired recall in IBS patients would be expected to attenuate rather than inflate observed associations, and the WURS was specifically designed and validated for retrospective adult use.

Childhood gastrointestinal symptoms were assessed with a single yes/no item, which does not capture severity or duration and should not be interpreted as a validated measure of early-life GI burden. Notably, IBS patients who retrospectively reported childhood GI problems did not differ from those who did not on any psychological or cognitive measure, which argues against these reports being driven primarily by current distress or recall bias.

The WURS was originally developed to assess childhood ADHD symptoms; however, the PCA-derived factors employed here reflect broader constructs of psychological distress, emotional dysregulation, and cognitive difficulties rather than ADHD-specific symptoms. Participants receiving pharmacological ADHD treatment were excluded, reducing the likelihood that elevated WURS scores reflect undiagnosed ADHD.

### Psychological distress and IBS

A positive association between current psychological distress and a retrospective account thereof was found across the whole sample. Although the correlations between childhood and adulthood were weaker for the emotional scales in IBS patients, they scored significantly higher on *WURS Factor 3 (Psychological Vulnerability and Distress)* than HCs, suggesting that they retrospectively remembered experiencing more psychological distress in childhood than the healthy controls. Such an early emotional vulnerability is in accordance with several animal and human studies, showing that early stress can alter gut permeability, induce a low-grade inflammation, and change microbiota composition later in life [11, 12, 46].

The absence of statistically significant WURS–HADS correlations in the IBS group after Bonferroni correction may be explained by several factors. First, the elevated levels of anxiety and depression in the IBS group, combined with the rarity of low HADS scores in this group, may have restricted the range of emotional symptom severity, thereby attenuating the ability to detect smaller associations with childhood behavioral factors. The Bonferroni correction is a conservative approach that controls strongly for Type I error at the cost of increased Type II error, and marginally significant associations may therefore have been suppressed. This finding also suggests that, in healthy individuals, psychological vulnerability may be less common in childhood, yet when present, it tends to be more persistent into adulthood than for patients with IBS [47]. Thus, based on our results, the experienced anxiety and depression that is highly prevalent in adult IBS patients may thus be a result of living with IBS symptoms rather than a vulnerability developed in childhood [48].

### Cognitive performance and IBS

The robust association between *WURS Factor 4 (Academic/Cognitive Challenges)* and adult cognitive outcomes in IBS patients, notably with a much weaker relationship for the HC group, was another important result. These findings invite several, not mutually exclusive, interpretations.

First, this pattern of association may reflect a stable neurocognitive trait that may predate IBS and co-occur across the lifespan, due to shared genetic, neurodevelopmental, or temperamental factors [49]. Supporting this, genome-wide association studies have identified shared genetic pathways between IBS and neurodevelopmental traits including cognitive ability and neuroticism, with specific genes such as CADM2 implicated in both IBS vulnerability and cognitive phenotypes [50–52].

Second, childhood cognitive difficulties may contribute to IBS vulnerability by acting as developmental risk factors. Academic struggles often involve repeated failure, reduced self-efficacy, and chronic stress, which can heighten stress responsivity and foster maladaptive coping [8]. Such pathways, through hypothalamic–pituitary–adrenal (HPA) axis dysregulation, autonomic imbalance, and amygdala hyperreactivity, can impair gut barrier integrity, activate immune responses, and disrupt microbiota composition [53, 54]. Even if these difficulties predate IBS, they may later be amplified by illness burden, as pain, fatigue, poor sleep, and unpredictable symptoms further compromise cognition [45]. In this view, early vulnerabilities may reduce adaptive capacity and interact with chronic illness to shape long-term outcomes.

Third, cognitive difficulties in IBS may also stem from early gut–brain dysregulation or shared origins in early life stress. Children predisposed to IBS may already experience abdominal discomfort, immune activation, or microbial imbalance during sensitive periods of brain development, potentially disrupting maturation of attention and memory networks [11, 55, 56]. At the same time, early life stress and adverse childhood experiences are linked to both cognitive impairments and functional somatic disorders [57, 58]. Stress during critical periods can alter prefrontal–limbic circuitry while sensitizing the gut–brain axis through increased permeability, immune dysregulation, and microbial shifts [53, 59, 60]. Together, this view suggests that early biological and psychosocial stressors converge to create enduring vulnerabilities that manifest as both cognitive difficulties and IBS symptoms. Overall, this wide range of interpretations warrants further studies.

### Clinical implications and treatment perspectives

Our findings emphasize that retrospectively identified childhood behavioral patterns and cognitive function in IBS are not only relevant for understanding etiology, but also hold important implications for how treatment and patient care can be tailored more effectively.

For patients, identifying connections between childhood behavioral tendencies and adult symptoms could provide a meaningful framework for understanding their condition. Many IBS patients struggle with the perceived legitimacy of their symptoms or feel stigmatized by a diagnosis that lacks clear biological markers [61]. A developmental perspective that acknowledges the interplay between early behavioral patterns and adult health may validate patients’ experiences and reduce self-blame.

It is important to note, however, that the association between childhood psychological vulnerability and adult anxiety and depression did not reach statistical significance within the IBS patient group after correction for multiple comparisons, which indicates that retrospective assessment of childhood behavioral patterns may be of specific value for understanding cognitive comorbidities in IBS.

For clinicians, our findings raise the possibility that a developmental perspective may complement standard psychological assessments in gastrointestinal evaluations. Current measures of anxiety and depression, as well as cognitive challenges, remain clinically important metrics for identifying patients who may benefit from integrated psychological care alongside conventional IBS management. The retrospective assessment of childhood behavioral patterns may offer additional context by capturing psychological vulnerability that predates IBS onset, though longitudinal studies are needed before such assessments could be recommended for routine clinical use. Future research might also examine whether early psychological intervention in children with IBS influences long-term symptom severity and treatment outcomes.

Ultimately, integrating developmental and behavioral dimensions into IBS assessment may refine our understanding of who is most at risk and when intervention is most likely to be effective.

## Conclusion

This study provides novel insight into how childhood behavioral characteristics, particularly psychological vulnerability and academic/cognitive challenges, relate to present cognitive and emotional function in IBS patients, showing distinct relationships between specific childhood characteristics and adult cognitive outcomes. The findings suggest that developmental trajectories, such as neurocognitive traits and early stress-related mechanisms, may contribute to the heterogeneity of IBS.

## Data Availability

The datasets used and/or analysed during the current study are available from the corresponding author on reasonable request.

## Abbreviations

IBS: Irritable Bowel Syndrome
HC: Healthy Control
DGBI: Disorder of Gut-Brain Interaction
GI: Gastrointestinal
B-BGM: Bergen Brain-Gut Microbiota
ACE: Adverse Childhood Events
WURS: Wender Utah Rating Scale
HADS: Hospital Anxiety and Depression Scale
RBANS: Repeatable Battery for the Assessment of Neuropsychological Status
IBS-SSS: IBS Severity Scoring System
PCA: Principal Component Analysis
ADHD: Attention Deficit Hyperactivity Disorder
